# Mechanical Overstimulation of Hair Bundles: Suppression and Recovery of Active Motility

**DOI:** 10.1371/journal.pone.0058143

**Published:** 2013-03-07

**Authors:** Albert Kao, Sebastiaan W. F. Meenderink, Dolores Bozovic

**Affiliations:** 1 Department of Physics and Astronomy, University of California Los Angeles, Los Angeles, California, United States of America; 2 California NanoSystems Institute, University of California Los Angeles, Los Angeles, California, United States of America; Tokai University, Japan

## Abstract

We explore the effects of high-amplitude mechanical stimuli on hair bundles of the bullfrog sacculus. Under *in vitro* conditions, these bundles exhibit spontaneous limit cycle oscillations. Prolonged deflection exerted two effects. First, it induced an offset in the position of the bundle. Recovery to the original position displayed two distinct time scales, suggesting the existence of two adaptive mechanisms. Second, the stimulus suppressed spontaneous oscillations, indicating a change in the hair bundle’s dynamic state. After cessation of the stimulus, active bundle motility recovered with time. Both effects were dependent on the duration of the imposed stimulus. External calcium concentration also affected the recovery to the oscillatory state. Our results indicate that both offset in the bundle position and calcium concentration control the dynamic state of the bundle.

## Introduction

Hair cells constitute the functional elements of the inner ear, as they transduce the mechanical vibrations into electrical signals that trigger action potentials in the auditory nerve [1], [2]. These cells derive their name from a specialized organelle on their apical surface that consists of a bundle of actin-packed stereocilia organized in a quasi-crystalline array. Along one axis within the bundle’s array, pairs of neighboring stereocilia are connected by filamentous tip links [3], [4], which are coupled to mechanically gated ion channels. Incoming stimuli deflect the stereocilia and hence increase the tension exerted on the tip links. A compliant element in series with the tip link, termed the gating spring, modulates the opening probability of the mechanically sensitive ion channels [5], [6]. The resulting influx of ions depolarizes the cell, leading to the release of neurotransmitter at the hair cell’s base.

Direct mechanical gating of transduction channels gives rise to a highly nonlinear response [7]–[9]. Furthermore, to maintain its extreme sensitivity in a dissipative aqueous environment, the inner ear must expend energy to amplify incoming stimuli [10]–[18]. Theoretical predictions based on nonlinear dynamics [19] have proposed that the hair cell achieves its sensitivity by operating in the vicinity of a bifurcation [20], [21]. If the bundle is near a stable fixed point, with an internal control parameter tuned close to a critical value, it can exhibit high amplification and sharp frequency selectivity [22]. A slight shift in the control parameter can render the fixed point unstable and lead to the appearance of a stable limit cycle. Spontaneous oscillations have been demonstrated in hair bundles of several species [23]–[28], and modeled in a number of studies as a limit cycle[29]–[31]. If an internal cellular mechanism could adjust the control parameter in response to the external environment, it would enable the hair cell to exert control over its sensitivity of detection. One of the signatures of tuning to or away from the critical point would be the appearance or suppression of spontaneous bundle oscillations.

Intense sound can cause a temporary elevation of hearing thresholds. These temporary threshold shifts (TTS) have been measured behaviorally in several mammalian species [32], [33]. Physiological measures have also demonstrated effects on the compound response of auditory nerve fibers [34], and cochlear microphonics [35]. These latter observations suggest that the mechanisms underlying TTS are peripheral, at the level of the hair cells. In this manuscript, we explore whether intense stimulation can induce a temporary shift of the hair bundle’s control parameter–as evidenced by a change in its dynamic state when it crosses the bifurcation point. Specifically, we search for suppression of spontaneous oscillations of the bundle following mechanical overstimulation.

We imposed high-amplitude deflections on individual hair bundles to explore the effects of overstimulation on cells that are alive and biologically functional, but isolated from neuronal feedback. Spontaneous oscillation was suppressed, the bundles remaining quiescent upon cessation of the stimulus. The quiescent interval was followed by recovery of the oscillatory regime. The effects of stimulus duration on the oscillatory activity, its suppression, and the subsequent recovery were quantified. We observed an initial offset in the resting position of the bundle, which increased with the duration of the imposed deflection. A slow negative drift gradually restored the natural position of the bundle, and allowed the return to oscillatory activity. The temporal profile of the slow motion is not fully captured by the known adaptation process [36], [37], but suggests the presence of other, slower, mechanisms.

Calcium has been shown to play an important modulatory role in the active process [38]–[42], affecting the rate of myosin-based adaptation [36] and exerting an effect on the channel-opening probability [43]–[46]. We explored the potential role of calcium as a control parameter governing the dynamic state of the hair bundle.

## Results

**Figure 1:**
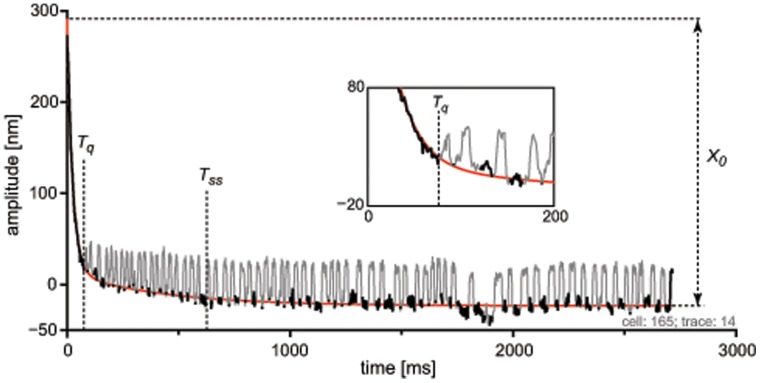
Example of hair bundle motility post-stimulus, with a diagram of the extracted parameters. The gray/black curve shows the position of the bundle, with motion towards the kinocilium being positive. An automatic routine detected bundle oscillations (*gray*) from which the onset time of the first oscillation (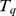) was determined. A sum of two exponentials (see Eq. 1) was fitted to the bundle’s position in the closed state (*black*), thus ignoring the oscillations. From the fit (*red*: 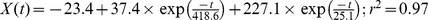), the time to reach steady state (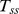) was calculated as the time for which its derivative first reached 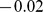 nm/ms. It was also used to calculate the bundle’s total offset (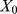), defined as the difference between the position at t = 0 and the end of the recording. The stimulus was a one-second DC offset in positive direction. The curve starts at t = 0, which corresponds to 5 ms after cessation of the stimulus.

Here, we report on recordings from 57 saccular hair cell bundles (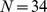 frogs) that exhibited spontaneous oscillations. Large (i.e.>1 µm) deflection of hair bundles in the positive direction–defined to be towards the tallest stereocilia, and corresponding to the preferential opening of the transduction channels–consistently led to a temporary suppression of the spontaneous oscillations following the stimulus (see Figure 1 for an example). In conjunction, bundles showed an offset in their position that decreased with time.

### Recovery of Bundle Position

**Figure 2:**
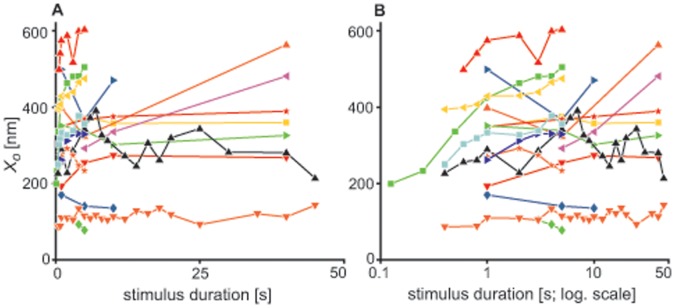
The initial offset induced in the position of the bundle depends on stimulus duration. (A) The bundle’s offset immediately following the cessation of the stimulus (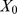) as a function of stimulus duration for 17 different cells. Cell are represented by different color/symbols. Results are shown for all the traces which yielded a good fit (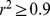 & 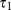 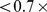 record duration). (B) Same as (A), but using a logarithmic abscissa.

Immediately following cessation of the stimulus, the average bundle position was at an offset with respect to its resting position. We calculated this offset (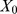) from the fitted exponential function (see Eq. 1) for all available traces. A significant range in 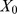 was observed across cells (∼100–600 nm). The size of 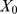 was positively correlated with the duration of the imposed deflection (Fig. 2) at shorter step durations (T<5 s). More variation in the offset was observed at longer deflections, with some cells showing plateaus or non-monotonic dependence on stimulus duration.

We note that the slow recovery of a bundle’s position cannot be accounted for by viscous drag alone. Based on estimates of the stiffness (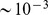 N/m) and drag coefficient (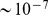 Ns/m) of a typical hair bundle [7], the return to its original position upon release from a 1 µm deflection should occur at sub-ms scales. This was verified by imposing brief pulse-like stimuli on the bundles (shown in Figure S1), which led to near-instant return to the original position.

**Figure 3:**
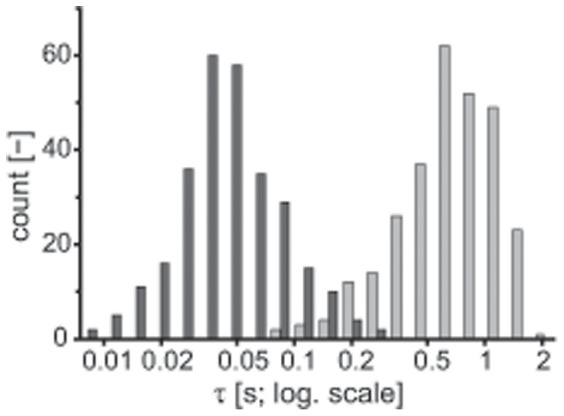
Post-stimulus adaptation of the resting position of the bundle exhibits at least two time scales. The slow component of the bundle movement following a prolonged deflection was fitted with the sum of two exponentials, yielding two time constants 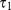 and 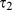, with 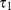
>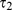. Good fits were obtained (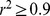 & 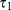 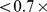 record duration) for 282 recordings (of 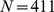). The two time constants are represented in the histogram by different shadings of gray.

The time course of the slow recovery is well described by two exponentials (Eq. 1). In all of the cases tested, this fit showed a statistically significant improvement over that of the single exponential (Student’s t-test, p<0.001). The calculated time constants (with 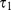 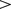 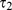) for the recorded traces (282 of 411 traces) are given in the form of histograms in Figure 3, and were (mean±std) 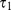 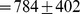 ms and 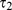 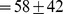 ms, respectively. We excluded traces for which the fit yielded 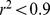 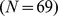, had 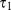 greater than 0.7 times the recording length 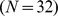, or could not be fitted uniquely 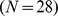. For the latter, bundle position often showed a very slow, nearly linear drift superimposed on the exponential decay.

**Figure 4:**
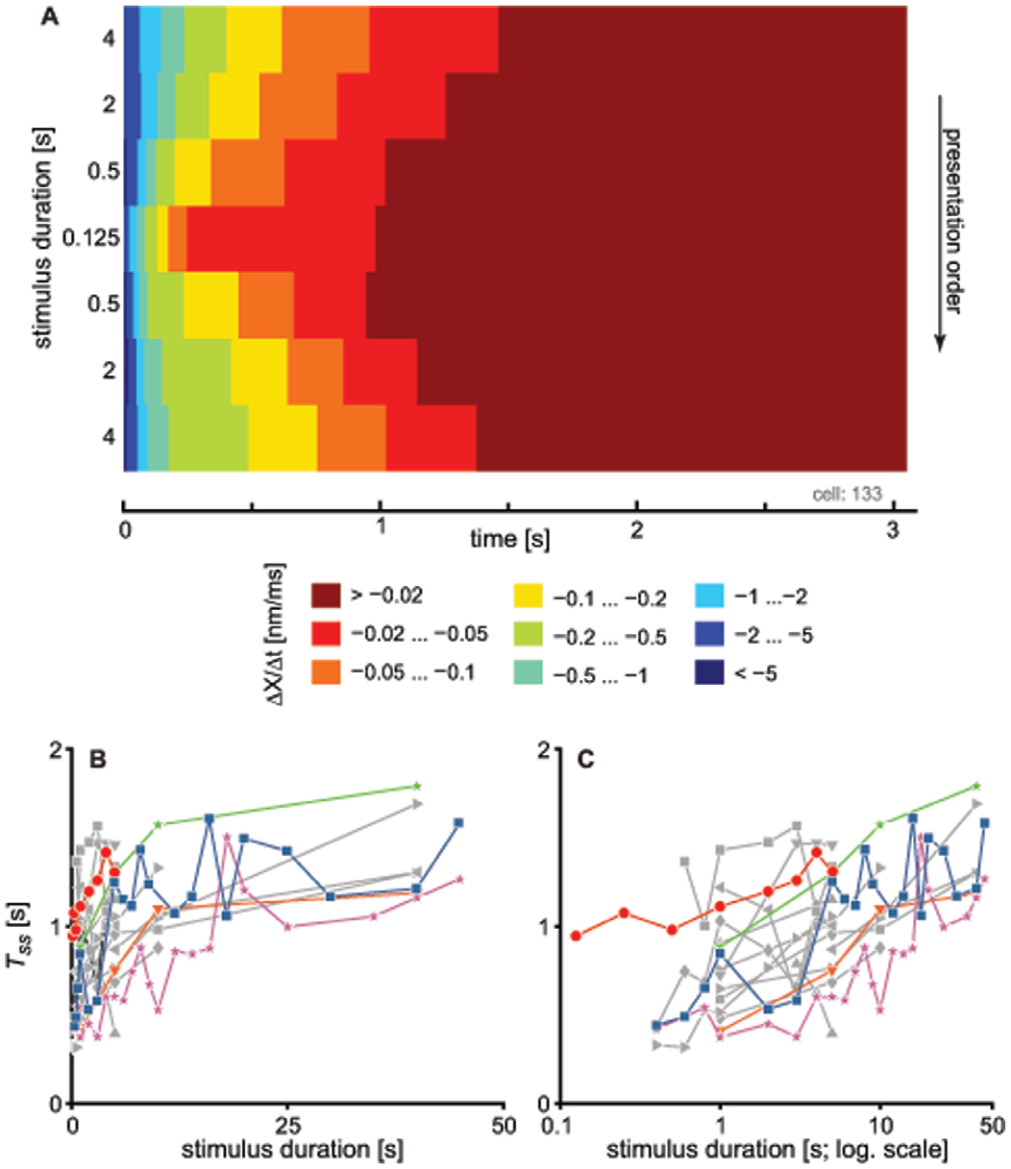
The time required by the bundle to return to its steady-state position depends on the stimulus duration. For a series of traces from a single cell, each recording was fitted with a sum of two exponentials. Derivatives of the fitted functions are represented in the form of a contour plot with slopes given in the legend (A). Rows correspond to different stimulus durations, with the recording order from top to bottom. We arbitrarily chose 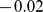 nm/ms as the threshold slope defining the steady-state. Similarity between the different contours indicates that the results do not critically depend on the selected criterion. (B) The time to reach steady-state (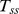) as a function of stimulus duration was determined for 17 different cells. These cells each had recordings for at least two different stimulus durations. Cells are represented by different symbols. The red line corresponds to the cell shown in (A). Five cells are displayed in color to guide the eye. The remaining cells are shown in gray with different symbols. (C) Semi-log plot of the data shown in (B).

As a measure of the dynamics of recovery, we determined the time required by the bundles to regain their steady-state positions. From the fitted function, this time (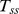, see Materials and Methods) is taken as the time when the derivative of bundle motion first reaches 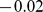 nm/ms. Figure 4A shows the contour plot of the derivative of hair cell motion for a series of recordings taken after step stimuli of fixed amplitude and varying duration. The plot demonstrates that the return to the steady-state position decreased with decreasing stimulus duration and increased when the stimulus was prolonged. Varying the threshold for 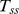 did not qualitatively affect its dependence on stimulus duration. Similar results were observed for 16 other hair cells (Fig. 4B,C): for short stimulus durations, 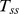 rises sharply, with a subsequent leveling off when stimulus durations exceed 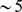 seconds.

### Recovery of Spontaneous Oscillations

**Figure 5:**
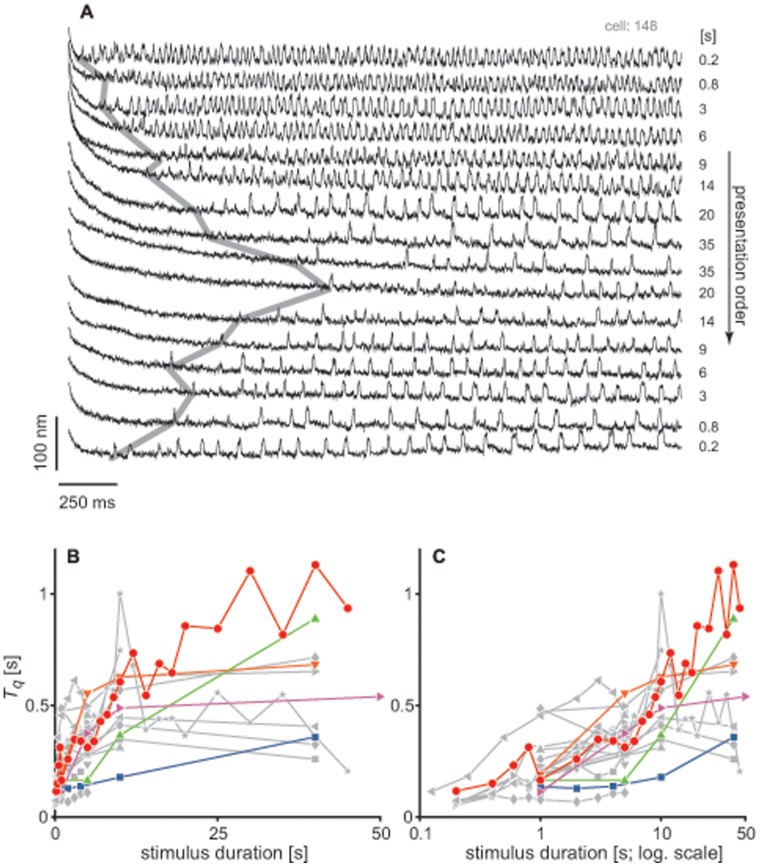
The time of the occurrence of the first bundle oscillation depends on stimulus duration. (A) Waterfall plot of a series of traces recorded from a single hair cell. Each trace was recorded following a stimulus of different duration (indicated on the right, in seconds), with the recording order from top to bottom. The thick gray line connects the start of the first bundle oscillation (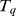) in each trace. (B) 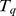 as a function of stimulus duration, for 20 cells for which recordings at a minimum of two different stimulus durations were obtained. Cells are represented by different symbols. The red line corresponds to the cell shown in (A). Five cells are displayed in color to guide the eye. The remaining cells are shown in gray with different symbols. (C) Same as (B), but plotted on a logarithmic abscissa.

**Figure 6:**
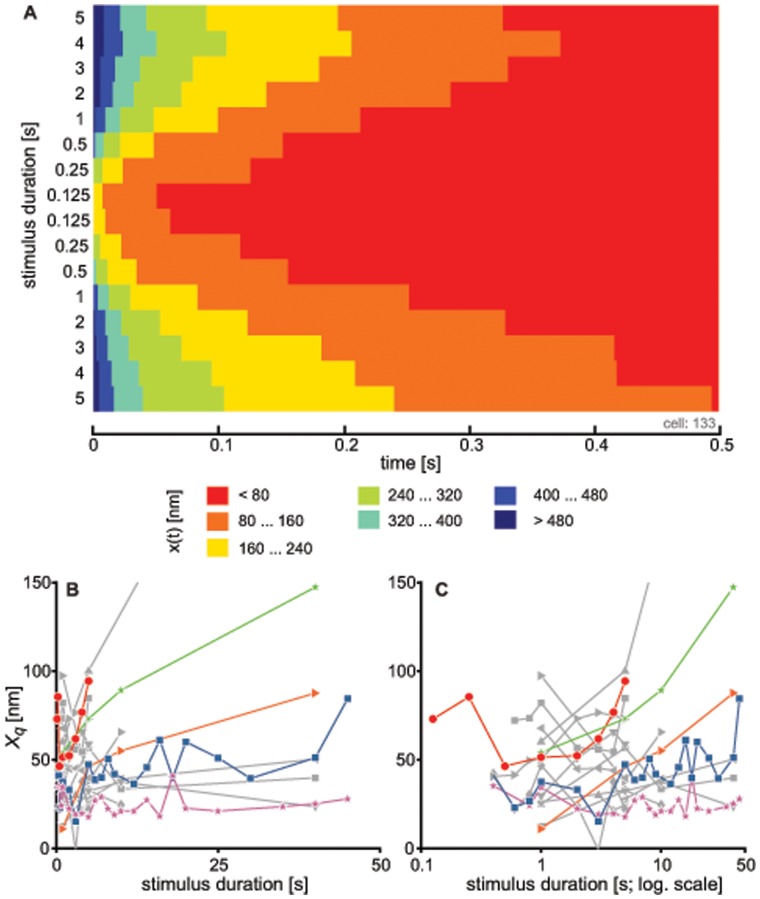
Offset in the position of the hair bundle position does not determine its dynamic state. For a series of traces obtained from a single cell, the bundle’s position, with respect to that at the end of the recording, is represented in the form of a contour plot (A). Different rows correspond to different stimulus durations, with the recording order displayed from top to bottom. (B) 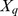 from 17 different cells for which recordings at a minimum of two different stimulus durations were obtained. Cells are represented by different symbols. The red line corresponds to the cell shown in (A). Five cells are displayed in color to guide the eye. The remaining cells are shown in gray with different symbols. (C) Same as (B), but plotted on a logarithmic abscissa.

All of the tested cells showed robust spontaneous bundle oscillations prior to stimulation. After the imposed deflection, the period over which the hair cells were quiescent varied systematically with the duration of the presented stimulus. As an example, Figure 5A shows a series of traces from a single hair cell, obtained following deflections of different duration. As the length of the stimulus increased, the time at which the bundle resumed spontaneous oscillation (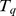) also increased. Note that subsequently decreasing the stimulus duration showed the opposite trend, indicating reversibility of the effect. While recovery was incomplete, with slower oscillations in subsequent recordings, the dependence of 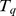 on stimulus duration (red line in Fig. 5B) remained robust. Figures 5B & 5C show the dependence of 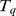 on the length of the imposed deflection for the 20 hair cells on which different stimulus durations were tested. A monotonic increase in the duration of the quiescent interval was observed, with a sharp rise from 0.1 to 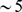 seconds of stimulus, and a more gradual increase with longer applied steps. Data could not be reliably obtained for durations above 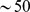 s, as damage was accrued. In conjunction with 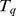, we also extracted the offset in the bundle’s position at the time of first oscillation (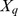). This parameter was likewise affected by stimulus duration, with a more sensitive dependence at short deflections (Fig. 6).

**Figure 7:**
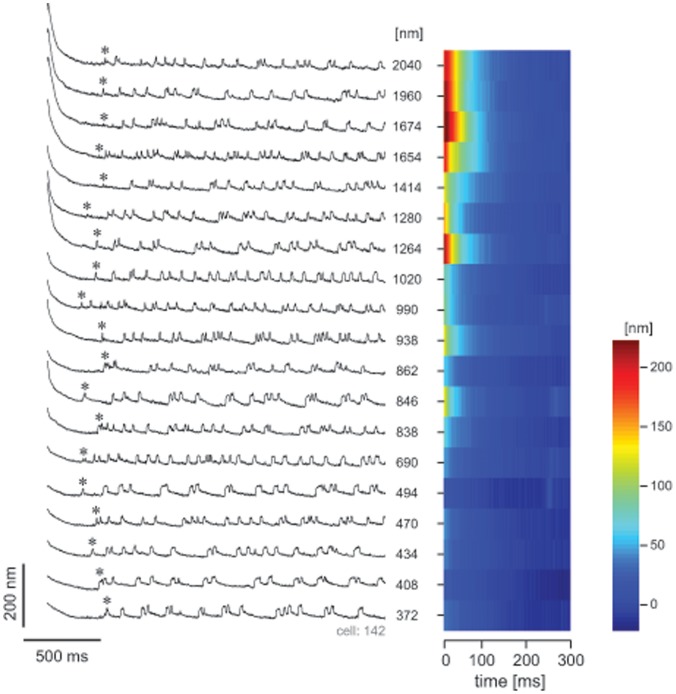
The duration of the quiescent interval 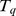 was not dependent on stimulus amplitude. Time-dependent traces of a hair bundle’s position after stimulation at various amplitudes, but at a fixed duration (5 s). The stimulus amplitudes are indicated on the right of the traces. The *asterisk* over each trace shows the time of first bundle oscillation, which does not vary with stimulus amplitude. On the right, same data are represented as a contour plot, with bundle position color-coded as indicated in the legend. The steady state position is affected only weakly by the imposed deflection.

We also quantified the dependence of quiescent interval on the magnitude of the stimulus. For 5 cells (82 recordings) a five-second stimulus was applied at various amplitudes (300–2100 nm) and the time to the first oscillation was determined. No systematic dependence of 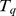 was observed over this range of amplitudes (Fig. 7).

### Effect of Calcium

**Figure 8:**
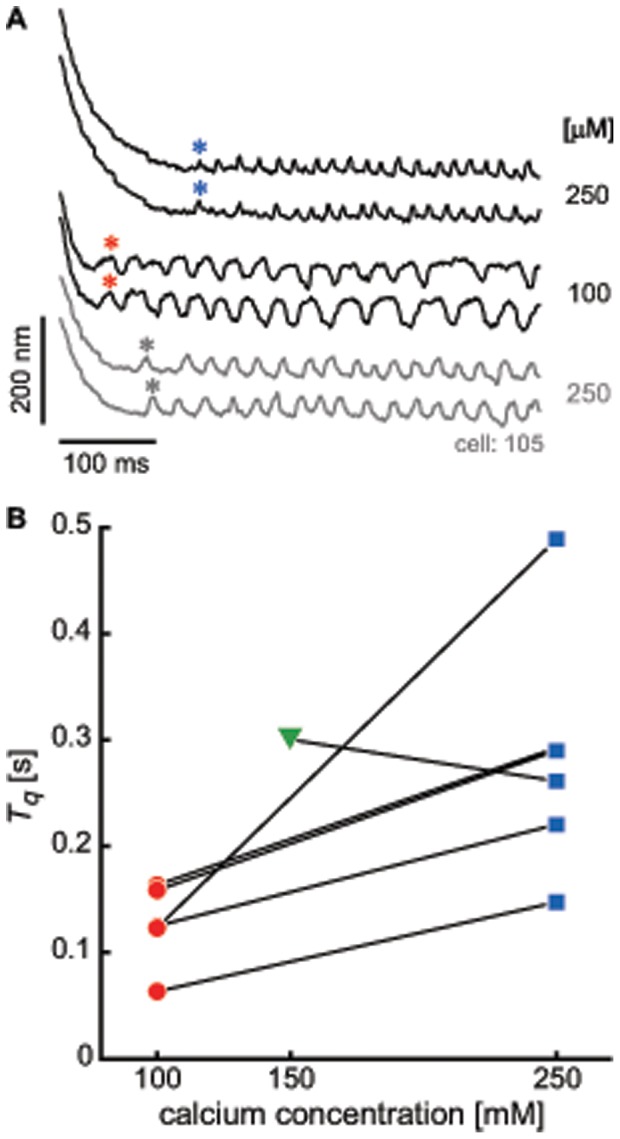
Different calcium ionic conditions on hair bundle recovery. Calcium concentration in the artificial endolymph was varied, as indicated to the right of the traces. (A) Concentration of calcium was first brought from 250 to 100 µM, which decreased 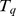 (*asterisk*). Subsequent raising the external calcium concentration back to 250 µM partially reversed the effect. The stimulus duration was 5 s. (B) Quiescent interval 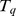 versus the concentration of calcium in the surrounding endolymph, obtained from 6 cells. Different colors/symbols denote different calcium concentration, and the green triangle showed the lone example that displayed an opposite trend as the other 5 cells.

As prior work has shown that calcium plays a modulatory role in active hair bundle motility [27], [38], [42], we explored its effects on the duration of the quiescent interval induced by prolonged deflection. Figure 8A displays examples of measurements in which the hair bundle was deflected for a fixed interval of time (5 seconds), under conditions of varying calcium concentration in the surrounding medium. Decreasing the external calcium concentration led to slower spontaneous oscillations, consistent with prior results in the field [27]. The quiescent interval (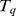) induced by the deflection was shorter, recovering more rapidly post stimulus cessation. Return to the original ionic conditions raised the frequency of oscillation; complete recovery of original oscillation was, however, seldom observed. Overstimulation in the presence of higher calcium concentration was detrimental to the hair cells, with increased variability and only a partial recovery upon return to baseline calcium conditions. We hence focus on effects of decreasing the external calcium concentration.

Figure 8B shows the intervals of induced quiescence (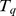) versus calcium concentration in the surrounding medium. Data are included only if obtained from hair bundles that displayed eventual recovery to the oscillatory state. Only recordings obtained while decreasing the calcium concentration were included in the plot. A dependence on external calcium concentration emerged in the time of the recovery to oscillation (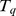).

As another measure of the effect of calcium, additional experiments were done in the presence of carboxyeosin diacetate (CEDA) in the external medium [42]. CEDA blocks the calcium extrusion pumps that are involved in maintaining the calcium concentration within the stereocilia. Inhibition of the extrusion pumps intensified the effects of overstimulation, prolonging the suppression of oscillation post stimulus (example shown in the Supplementary material, Fig. S2). The effect was however convolved with irreversible rundown of the active bundle motility in the presence of the blocker.

## Discussion

To assess the effects of overstimulation on the dynamic state of a hair bundle, we imposed prolonged high-amplitude deflections and measured the subsequent recovery. Our measurements revealed two main effects, an induced offset in the resting position of the bundle and temporary suppression of the innate oscillation. Both effects could persist for seconds after the stimulus and showed a dependence on the stimulus duration.

### Recovery of Bundle Position

Relaxation of the hair bundle to its equilibrium position following cessation of the stimulus exhibited a complex temporal profile. Fitting the data with two exponentials (Eq. 1) allowed us to extract the timescales characterizing the motion with no *a priori* assumptions as to the biochemical identity of the adaptation mechanisms. The excellent fit of this function to the data implies the existence of two processes that operate on very different time scales.

Multiple adaptation mechanisms have been demonstrated in hair cells of the inner ear. Myosin motors enable a tension-sensitive adaptation, by climbing and slipping along the actin core of the stereocilia [36]. The shorter time constant that we observed (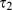 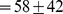 ms; Fig. 3) is consistent with that expected for myosin-based adaptation in the bullfrog sacculus. The initial slope of the recovery, however, indicates climbing speeds significantly higher than those previously reported for myosin 1C [27]. The initial recovery therefore may reflect signatures of a fast adaptation process [46], also mediated by the myosin motors [28] or caused by an additional relaxation element [44]. For the longer time constant (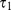 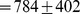 ms; Fig. 3), we recently proposed the existence of a variable gating spring with a calcium-dependent stiffness [47]. This element was hypothesized to exhibit dynamics that are slow with respect to the myosin-based adaptation. The slow dynamics of recovery measured in these experiments are consistent with those of the variable gating spring proposed in the model. Across the recordings, the two time constants were correlated (correlation coefficient = 0.47), indicating that the two mechanisms are not independent.

As the slower adaptation mechanism was affected by calcium, its time constant may reflect the relatively slow dynamics of calcium pumps within the stereocilia. During stimulus presentation, the transduction channels are kept in a preferentially open state for a prolonged period of time, during which calcium could accumulate within the stereocilia. After cessation of the stimulus, the slow extrusion of calcium by these pumps would gradually restore the concentration to the internal resting level. The accumulated internal calcium could also affect myosin-based adaptation, an internal relaxation element, and opening probability of the ion channels, thus affecting the faster time scale as well.

### Tuning of the Dynamic System

Apart from the observed relaxation of the hair bundle’s position, the second striking effect of high-amplitude mechanical deflection was to induce a temporary crossover from the oscillatory to the quiescent state. Theoretical models of hair bundle motility have proposed that it can exhibit a stable limit-cycle oscillation, or operate in a regime where it amplifies but remains quiescent in the absence of input [20], [22]. The bifurcation between these regimes is under control of an internal parameter, which has been proposed to be modulated by a feedback mechanism [21]. In a recent study [48], we modeled hair bundle dynamics as a system poised near a subcritical Hopf bifurcation, with bistable switching between oscillation and quiescent state. A feedback equation on the control parameter maintains it near the bifurcation in the oscillatory regime. In the model, a strong stimulus can shift the control parameter across the bifurcation point, which leads to suppression of spontaneous oscillation. The feedback equation then describes the subsequent recovery of the control parameter to its original value, returning the bundle to its oscillatory state.

The time required for recovery to the oscillatory state (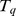) was observed to be sensitively dependent on the duration of the applied stimulus (Fig. 5). This finding is consistent with the proposed notion of dynamic feedback on the control parameter. As longer stimuli would lead to further detuning of the parameter, the system would exhibit a slower subsequent recovery. Stimulus duration also affects the offset in the bundle position (Fig. 2), which constitutes one of the candidates of the control parameters [21], [22]. Offset alone, however, does not explain all of the experimental observations. First, variation in the stimulus amplitude, which results in varying initial offsets, has little effect on the timing of the recovery of bundle oscillations (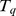; Fig. 7). Second, variation in the external calcium concentration (Fig. 8) or the application of calcium pump blockers (Fig. S2) exert an effect on 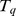, without significantly changing the dynamics of position recovery. The data indicate that in addition to the induced offset, calcium plays a direct role in modulating the dynamic state of the hair bundle. Extensive literature exists on the importance of calcium feedback in the dynamics of individual hair cells [5], [6], [21], [22], [27], [28], [45]. Our experiments are consistent with this prevailing view.

We propose that calcium and offset in the bundle position both exert an effect on the dynamic state of the bundle. The effects of these two parameters are convolved: an influx of calcium into the bundle during the stimulus enhances the slippage of myosin motors and thus induces an offset in the resting position of the bundle [36]. Calcium could also exert a direct effect on the stiffness of the bundle by binding to the proposed variable gating spring [47], thus affecting bundle’s offset. Conversely, imposing an offset changes the opening probability of the transduction channels [11], which affects the internal calcium concentration. Subsequent to the imposed deflection, Ca^2+^-extrusion pumps reduce the internal concentration [49]. This decreases the induced offset as myosin motors restore the original resting position of the bundle.

Tuning of the hair bundle to the vicinity of a bifurcation may explain the remarkable sensitivity displayed by endorgans of the inner ear. Temporary detuning of the control parameter would in turn result in a brief decrement of the system’s sensitivity. Transient changes in the sensitivity have been observed experimentally *in vivo*, including shifts of auditory threshold (TTS) [32], [33] and changes in the compound response of auditory nerve fibers [34] and cochlear microphonics [35]. Tuning of the dynamic state of a hair bundle without involving delays inherent in signaling to the brain and back would allow a rapid adaptation in its sensitivity. A peripheral gain control could enhance the robustness of the hair cell and thus protect it against accrued damage.

## Materials and Methods

### Biological Preparation

Prior to performing experiments, all animal-handling protocols were approved by the UCLA Chancellor’s Animal Research Committee (Protocol Number ARC 2006-043-13C) in accordance with federal and state guidelines. Experiments were performed on *in vitro* preparations of the sacculus [50], [51], excised from the inner ear of the North American bullfrog (*Rana catesbeiana*). The preparations were mounted in a two-compartment chamber, with hair cells exposed to artificial perilymph and endolymph on the basal and apical sides respectively. The solutions were made to mimic the ionic conditions in the sacculus; for perilymph, it contained (in mM) 110 Na^+^, 2 K^+^, 1.5 Ca^2+^, 113 Cl^−^, 3 d-glucose, 1 sodium pyruvate, 1 creatine, 5 HEPES; and for endolymph: 2 Na^+^, 118 K^+^, 0.25 Ca^2+^, 118 Cl^−^, 3 d-glucose, 5 HEPES. Both solutions were oxygenated for 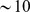 minutes prior to use. The overlying otolithic membrane was removed from the epithelium with an eyelash tool following a 7–8 minute enzymatic dissociation with 15 µg/mL Collagenase IV (Sigma Aldrich). Active spontaneous oscillation was observed in hair bundles after decoupling from the membrane, and could be maintained for several hours post dissection.

### Detection of Hair Bundle Motility

Preparations were imaged in an upright optical microscope (Olympus B51X) with a water immersion objective (20X, 0.95 N.A.) and illuminated with a X-Cite 120W halogenide lamp. Images were further magnified to ∼400X and projected onto a high-speed Complementary Metal Oxide Semiconductor (CMOS) camera (Photron FASTCAM SA1.1). The motion of the hair bundles was tracked with software written in MATLAB (The MathWorks). A horizontal line scan was taken through the image in each frame, and a Gaussian distribution is fit to the intensity profile of the bundle to extract the center position. To improve the signal-to-noise ratio, 11–15 vertically adjacent pixel rows were tracked and averaged. Time-dependent traces of the movement were then obtained by plotting the extracted center position of the bundle for each frame of the record. The noise levels in these recordings were ∼3–5 nm.

### Mechanical Stimulation

To detune the cells away from their natural dynamic state, we applied prolonged mechanical overstimulation. Glass fibers were pulled with a micropipette puller (P97, Sutter Instruments), with an additional rod ∼2–3 µm in diameter fabricated at a 90 degree angle using a home-built puller. The fibers were mounted on a piezoelectric stimulator (P-150 Piezo Jena) and positioned in the vicinity of the hair bundle with micro-manipulators. Step signals were generated with a function generator (Tektronix; model AFG 3022), low-pass filtered with an eight-pole Bessel filter at 100 Hz (Krohn Hite; model 3382), and sent to the piezoelectric amplifier. CMOS camera recording was synchronized with cessation of stimulus. Deflection imposed on the bundles was measured from the video recordings by subtracting the bundle position under deflection from the original position. Only records where no adhesion was observed between the probe and the bundle were included in the analysis. To allow hydrodynamic effects induced by the probe to settle, we allowed 5 ms to elapse post its withdrawal and plotted the subsequent response. A video example of the stimulation and subsequent recovery is given in Video S1.

### Fitting Procedures and Analysis

To minimize the effects of cell rundown, we only included those hair cells in the analysis hat showed robust bundle oscillations of more than 4 Hz prior to the first stimulus protocol. In addition, the spontaneous bundle motility had to recover to more than 3 Hz, either immediately following a particular stimulus, or at the end of the last stimulus protocol.

Several parameters were extracted from the time-dependent traces 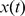 (see also Fig. 1) using software implemented in MATLAB. First, the trace was bandpass-filtered (0.5–150 Hz), and candidate oscillations were identified as large (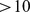 nm) excursions towards the kinocilium. Next, the maximum slopes leading to and following after each candidate oscillation were calculated. If the absolute value of both slopes exceeded 1 nm/ms (i.e. 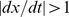 nm/ms), the excursion was considered an oscillation of the bundle.

To extract the characteristic times of the recovery of the bundle in the closed-channel position, the identified oscillations were removed from the trace, and the remaining data were fitted to the function:
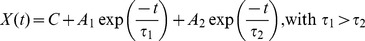
(1)


Besides providing the two time constants (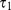, 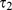), the fitted function was used to calculate the time of recovery to the steady state (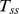; the first moment 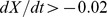 nm/ms after cessation of the stimulus), and the initial offset in the bundle position after stimulation (re. the bundle’s postion at the end of the recording, 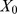 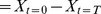, where *T* is the duration of the record). Based on the detected oscillations, we define 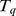 as the starting time of the first oscillation, with 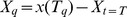 being the corresponding position of the bundle.

### External Calcium Concentration and Extrusion Blockers

Mechanical deflection was applied at a fixed amplitude and duration, under different ionic conditions. To increase the calcium concentration between recordings, small quantities of high-calcium endolymph were added to the solution bathing the apical surface of the preparation. To lower the calcium concentration, the endolymph in the top compartment was completely replaced by endolymph at the desired calcium concentration using a peristaltic pump. Equivalents of 1 mL of the top solution were exchanged.

For one set of experiments, we blocked the calcium extrusion pumps with carboxyeosin diacetate succinimidyl ester (CEDA; Sigma Aldrich). The reagent was dissolved in 0.2% dimethyl sulfate oxide (DMSO), then diluted to concentrations of 1–10 µM in endolymph.

## Supporting Information

Figure S1 **Hydrodynamic effects on recovery are fast.** A spontaneously oscillating hair bundle was mechanically stimulated every 500 ms with a large (>1 µm), and brief (5-ms) pulse (*asterisks*). Such transient, large offsets led to fast recovery indicating that hydrodynamic effects are negligible in the observed slow recovery that follows longer stimulations. In the analysis of all data, the first 5 ms following each stimulus are excluded, to further minimize the hydrodynamic effects.
(TIF)

Figure S2
**Blocking calcium extrusion pumps affects the recovery of spontaneous bundle oscillation.** The top two traces are examples of hair bundle motion in the absence of the extrusion pump blocker CEDA for one cell; *asterisks* over the traces indicate the occurrence of the first bundle oscillation. The bottom two traces were recorded from the same cell after the addition of 1 µM CEDA, a known blocker of the calcium extrusion pumps, to the external solution. For all recordings, stimulus parameters were unaltered between the recordings. We consistently observed an increase in the time to first bundle oscillation (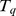) with the addition of CEDA into the solution.(TIF)

Video S1
**Suppression and subsequent recovery of spontaneous bundle oscillations following stimulation.** The top half of the video gives a top down view of one hair bundle and the stimulus probe. Scale bar corresponds to 2 µm. In the video, the one-second stimulus ceases at t = −5 ms, and tracking of the bundle’s position starts at t = 0 (bottom half of the video). The original video was obtained at 1000 fps, but shown here downsampled (1 of every 10 frames), and slowed 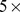.(AVI)
