# Supplementary figures and images for: Mechanical Overstimulation of Hair Bundles: Suppression and Recovery of Active Motility
Source: PLoS One. 2013 Mar 7;8(3):e58143. doi: 10.1371/journal.pone.0058143 (PMC3591416; doi:10.1371/journal.pone.0058143)

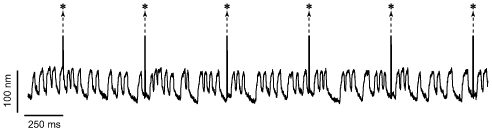

Supplement: Figure S1 — Hydrodynamic effects on recovery are fast. A spontaneously oscillating hair bundle was mechanically stimulated every 500 ms with a large (>1 µm), and brief (5-ms) pulse (asterisks). Such transient, large offsets led to fast recovery indicating that hydrodynamic effects are negligible in the observed slow recovery that follows longer stimulations. In the analysis of all data, the first 5 ms following each stimulus are excluded, to further minimize the hydrodynamic effects. (TIF) [file pone.0058143.s001.tif]

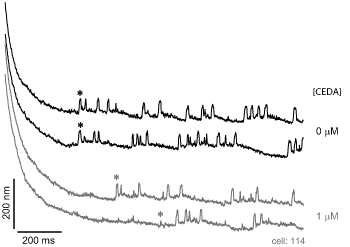

Supplement: Figure S2 — Blocking calcium extrusion pumps affects the recovery of spontaneous bundle oscillation. The top two traces are examples of hair bundle motion in the absence of the extrusion pump blocker CEDA for one cell; asterisks over the traces indicate the occurrence of the first bundle oscillation. The bottom two traces were recorded from the same cell after the addition of 1 µM CEDA, a known blocker of the calcium extrusion pumps, to the external solution. For all recordings, stimulus parameters were unaltered between the recordings. We consistently observed an increase in the time to first bundle oscillation () with the addition of CEDA into the solution. (TIF) [file pone.0058143.s002.tif]
